# Insights into climate variability of the meteorological records from a background monitoring station: the Giordan lighthouse, Gozo

**DOI:** 10.12688/openreseurope.19099.2

**Published:** 2025-07-15

**Authors:** James Ciarlo`, Ryan Vella, Martin Saliba, Raymond Ellul, Alfred Micallef, Erika Coppola, Aaron Micallef, David Mifsud

**Affiliations:** 1 Institute of Earth Systems, University of Malta, Msida, Malta; 2 Earth System Physics Section, Abdus Salam International Centre for Theoretical Physics, Trieste, Friuli-Venezia Giulia, Italy; 3 Atmospheric Chemistry Department, Max Planck Institute for Chemistry, Mainz, Germany; 4 Institute for Atmospheric Physics, Johannes Gutenberg University Mainz, Mainz, Germany; 5 Department of Geosciences, University of Malta, Msida, Malta; 6 Monterey Bay Aquarium Research Institute, Moss Landing, California, USA

**Keywords:** Short-Term Climate Variability, Meteorological Records, Background Monitoring Station, Giordan Lighthouse, Gozo

## Abstract

**Background:**

The Maltese islands are subject to substantial climate variability, with implications for ecosystems and human activities. This study leverages a 26-year dataset from the Giordan Lighthouse Background Monitoring Station (GL) on the island of Gozo to analyse short-term climate variability and its alignment with broader regional tendencies.

**Methods:**

Hourly meteorological data collected from 1997 to 2022, including wind speed, wind direction, air temperature, relative humidity, and air pressure, were analysed. The study examined diurnal and annual cycles, probability distribution functions, and climate indices to characterise local climate dynamics. Qualitative comparisons were made to existing findings based in Malta to corroborate the results.

**Results:**

The analysis revealed pronounced seasonal variability in all parameters. Rising air temperatures were detected, consistent with regional warming tendencies. Humidity and wind conditions showed seasonal shifts aligning with observations from other regional monitoring stations. The high-resolution dataset also captured fine-scale temporal patterns, reinforcing the critical value of localised, long-term meteorological monitoring for understanding climatic shifts.

**Conclusions:**

This study underscores the value of long-term meteorological datasets in detecting climate variability, including a clear warming pattern and seasonal shifts in temperature, humidity, and wind conditions. Continuous monitoring and improved data reliability are essential for enhancing climate assessments and supporting effective adaptation strategies in the Maltese Islands.

## Introduction

The Maltese Islands, situated in the heart of the Central Mediterranean, are characterised by a distinct climate with hot, dry summers and mild, wet winters. The climate of the Maltese Islands, shaped by its geographic location and proximity to the North African coast, has a history of scientific investigation ( England *et al.*, 2010; Jahan *et al.*, 2022; Wadsworth, 1928). Notably, local research ( Azzopardi, 2016; Galdies, 2010; Galdies, 2011; Galdies, 2012; Galdies, 2022; Hyde, 1955) has shed light on various aspects of Maltese climate. These studies contribute to a foundation of knowledge and understanding of the Maltese climate.

According to data collected between 1961 and 1990 from the Malta International Airport in Luqa, Malta ( Galdies, 2010; Galdies, 2022), the seasonal cycle is defined by monthly averages of approximately 12 °C in January and February, and peak annual temperatures between July and August with averages above 25 °C. At their most extreme, temperatures have varied between 1.4 °C and above 43 °C ( Galdies, 2011; Galdies, 2022). Over recent decades, the average temperature has been steadily increasing, reaching an anomaly of 1.5 °C higher than averages in 1952 ( Galdies, 2022). Relative humidity (RH) also varies seasonally, with an average of ~79% in winter, and ~69% in summer ( Galdies, 2011; Galdies, 2022). Furthermore, a recent report ( Galdies, 2022) has noted a gradual decrease in average RH on the island of 0.8% per decade, a phenomenon that can be attributed to the observed warming, and also potentially to the aridification of the region ( Doblas-Reyes *et al.*, 2021; Gutiérrez *et al.*, 2021; Ranasinghe *et al.*, 2021). The near-surface winds (measured at 10 m above ground) being predominantly north-westerly (centred at 315°), vary with a winter average of 5.25 m s-1 (10.2 knots) in winter to 3.54 m s-1 (6.9 knots) in summer ( Galdies, 2022; Wadsworth, 1928). These studies also reveal that the Maltese islands may still be undergoing terrestrial stilling ( McVicar *et al.*, 2012; Vautard *et al.*, 2010), a phenomenon which may be in the process of reversal ( Zeng *et al.*, 2019).

However, there remains a need for continuous monitoring and analysis of climate variability, particularly in the face of ongoing global rapid climate change. The Giordan Lighthouse Background Monitoring Station (henceforth referred to as GL), established in 2000 and accredited as a Global Atmosphere Watch (GAW) station in 2005 ( Ellul & Saliba, 2005), serves as a crucial site for this purpose. The station's suite of instruments, specifically chosen for their accuracy in harsh coastal environments, has been collecting high-resolution meteorological data for over two decades.

This study utilises the dataset from the GL to assess climate variability within this background monitoring station by comparing it with findings of existing research within the Maltese islands. While past research with GL data was primarily focused on wind and air quality ( Azzopardi, 2016; Ellul & Saliba, 2005; Matasović *et al.*, 2023), we aim to provide a more comprehensive analysis of climate variability at the GL by:

- reporting on any missing data gaps and outliers within the time series;
- delving into meteorological parameters in addition to winds, allowing for a more holistic understanding of the climate system in the region;
- assessing climate variability within annual and diurnal cycles;
- exploring the variation of extreme indices derived from these parameters, providing insights into the frequency and intensity of extreme weather events at the GL.

## The giordan lighthouse background monitoring station

### History

The GL was constructed when the Maltese Archipelago was a British Colony. It started operating on 15 March 1853 ( D’Amico *et al.*, 2024). The purpose of the GL was to act as a reference point for vessels crossing the Strait of Sicily and those approaching the Maltese islands, thus becoming an important landmark for navigation. As vessels were equipped with transponders and navigation devices, the physical presence of the GL was no longer needed for navigation.

After this time, the purpose of the GL shifted to provide a local and international scientific contribution. A joint Maltese-German atmospheric pollution research programme was launched on 7 December 1996 to monitor atmospheric pollution over the Maltese islands and in the Central Mediterranean ( D’Amico *et al.*, 2024; Ellul & Saliba, 2005). Since the establishment of this research programme, several meteorological parameters have been continuously measured and recorded, including wind speed and direction, air temperature, relative humidity, and atmospheric pressure. The resulting long-term dataset contributed to numerous air quality studies ( Azzopardi *et al.*, 2013; Cristofanelli *et al.*, 2015; Kalabokas *et al.*, 2008; Nolle *et al.*, 2002; Saliba *et al.*, 2008; Saliba *et al.*, 2021; Sofen *et al.*, 2016; Tenkanen *et al.*, 2021; Tsuruta *et al.*, 2017), while the meteorological dataset forms the core of the study being reported here.

### Instrument and data collection

**Figure 1.**
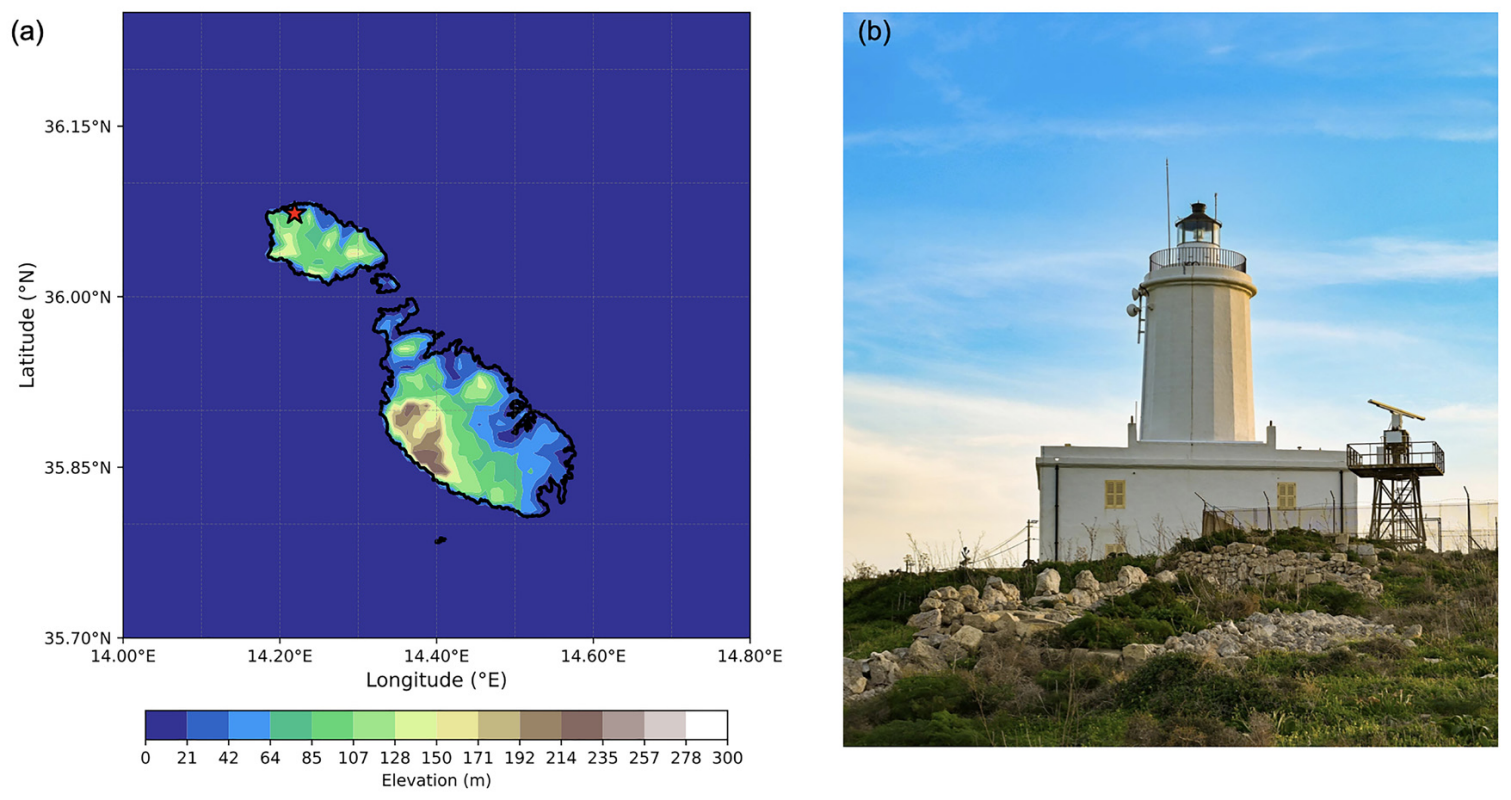
( **a**) Elevation of Malta and Gozo with the station position, and ( **b**) a photo of the GL monitoring station.

The GL is located on Ġurdan Hill overlooking the village of Għasri on the northwest coast of the island of Gozo ( Figure 1). Its base is 163 m above mean sea level and approximately setback by 800 m from the coastline. The Lighthouse is 21 m in height and has geographic coordinates 36° 04’ 24” N and 14° 13’ 08” E. The instruments used at the GL include a cup and vane anemometer (Lambrecht model 14512, replaced by Lufft model WS200 on 28th July 2021, installed at the top of the lighthouse) to measure wind speed and direction, a Vaisala HMP60 probe (installed approximately 4 m above the base level) to measure relative humidity and air temperature, and briefly, between 2002 and 2008, the Vaisala PTB110 was installed at approximately 167 m above mean sea level, to measure air pressure. All variables are recorded at one minute interval (instantaneous), and then averaged to an hourly resolution ( Saliba & Micallef, 2025). This study provides an analysis of the meteorological data (variables and instrument information are summarised in Table 1) collected from this station over a 26-year period, ranging from 1997 to 2022, with an hourly temporal resolution.

**Table 1.** Abbreviations and descriptions of meteorological variables measured at the Giordan Lighthouse Background Monitoring Station. Lambrecht instrument for WS and WD was applicable up to 2017, and the Lufft instrument from 2020 onwards. FS = Full Scale (range provided in parenthesis), RMSE = Root Mean Square Error.

Abbreviation	Full Name	Units	Instrument	Uncertainty
WS	Wind Speed	m s ^-1^	Lambrecht 14512 Wind anemometer	± 2% FS (0 - 35 m/s)
			Lufft WS200 Smart weather sensor	±0.3 m/s
WD	Wind Direction	°	Lambrecht 14512 Wind anemometer	± 1% FS (0 - 360°)
			Lufft WS200 Smart weather sensor	<3° RMSE, >1.0m/s
RH	Relative Humidity	%	Vaisala HMP60 Probe	±3%
AT	Air Temperature	°C	Vaisala HMP60 Probe	±0.6 °C
DT	Dew Point Temperature	°C	*Calculated using Equation (8) in Lawrence (2005)*	*±5.7 °C*
AP	Air Pressure	hPa	Vaisala PTB110 Barometer	±0.3 hPa

The long-term nature of data collection inevitably leads to occasional technical issues. Data outside the sensor's measurement range is automatically flagged as invalid. The radiation shield housing the Vaisala probe is inspected every two weeks, and both the shield and probe are carefully cleaned when there is presence of light dust. Periodically, both the AT and RH readings are compared with those from nearby stations overseen by the Malta Meteorological Office. The Lambrecht wind anemometer was serviced once a year by putting three drops of fine oil in the head. No further calibration and maintenance was required as per the manufacturer's instructions (including for the Lufft anemometer).

Despite these regular checks, occasional irregular readings may still occur. Here, we discuss specific instances where data points were removed due to problems identified with the instruments. Notably, eight data points were identified where the pressure was around 750 hPa, with a change of well over 200 hPa from the previous and following time-steps. Additionally, a data gap occurred between September 2003 and December 2004 because the probe was not operational during that time.

A WS data gap is present between 26th September 2017, and 31st December 2020, which originated due to a malfunction in the original cup and vane anemometer (Lambrecht model 14512). To restore reliable data collection, the instrument was replaced with a Lufft model WS200 anemometer. Additionally, a separate period from 21:00 on 13th April 2017 to 06:00 on 5th July 2017 was excluded, as the values remained constant at 6.4 m s-1, due to a sensor malfunction, which was later resolved.

Although the Vaisala HMP60 probe is designed to measure RH within a 0–100% range, certain readings in this dataset fell outside this operational window. Notably, a value of 1766% was recorded at 01:00 of 13th April 2000 and a value of -462% recorded at 07:00 of 7th December 1999. All outliers exceeding the sensor's range were removed from the dataset. Additionally, instances where RH suddenly dropped to 0% (or close to it), such as at 04:00 on 3rd June 2019 and between 03:00 and 05:00 on 19th June 2019, were also excluded. In total, 1006 data points were removed due to these malfunctions.

Similarly, some AT readings were characterised by large and sudden deviations (e.g., 19°C to 47°C or above) in temperature compared to the nearest recorded values. The data points between the start and end of such activity were excluded from the dataset. The specific instances of these anomalous readings, which totalled 231 data points, included: 10:00 on 1st August 2016; 07:00 on 2nd August 2016; 09:00 to 18:00 on 15th June 2018; 21:00 on 2nd May to 01:00 on 16th May 2019; 16:00 on 24th May to 11:00 on 25th May 2019; 08:00 to 10:00 on 2nd June 2019; 05:00 to 07:00 on 3rd June 2019; 03:00 on 6th June 2019; and 08:00 on 11th June 2019. All these anomalous readings were removed from the analysis dataset but preserved separately in the Supplementary Material.

### Data coverage

**Figure 2.**
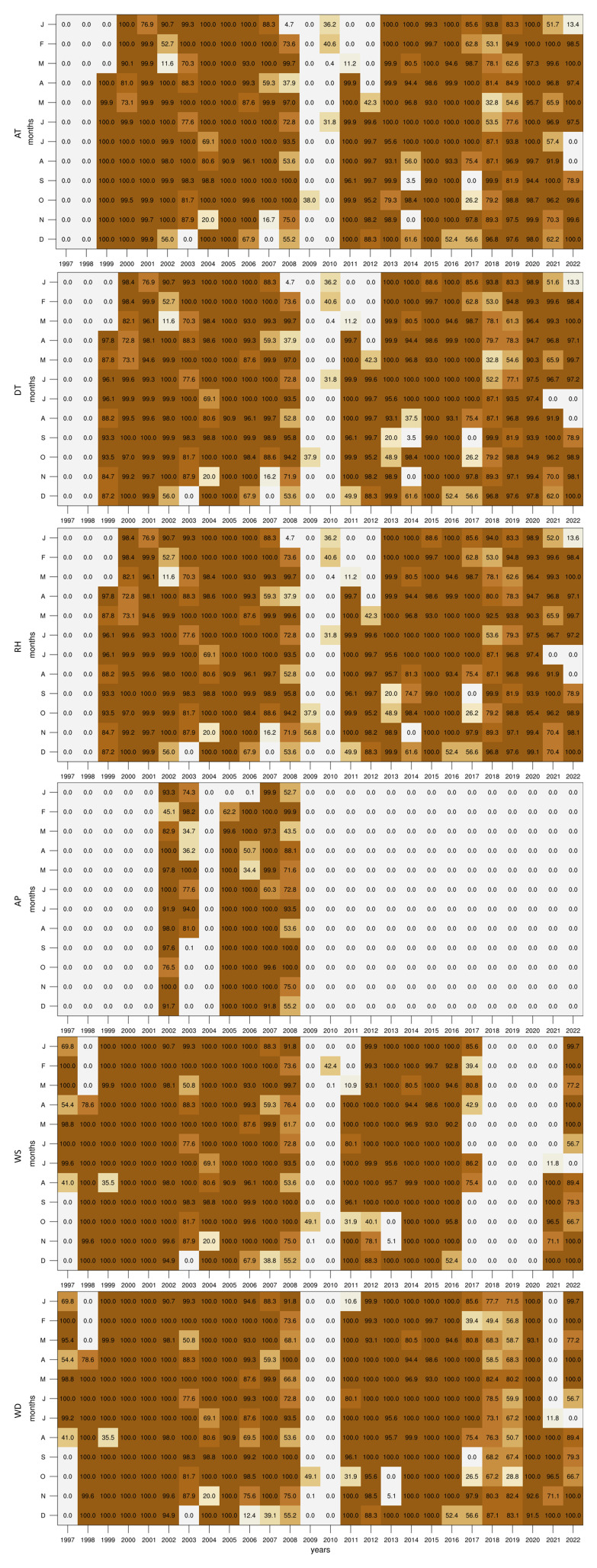
Percentage of valid data points for each month and variable in the dataset.

In this study, we begin by assessing the completeness of the dataset over the 25-year period. Specifically, we calculate the percentage of data available for each month across all six variables. This preliminary analysis is crucial for identifying periods of missing data, which could impact the validity of subsequent analyses and interpretations. The results of this initial assessment are visualised in Figure 2, which provide a clear overview of the data availability across different months and years.

Although the dataset starts in 1997, only WS and WD contain data within the first two years, as the GL station was still in the early stages of setup. All variables are missing data in 2009 and 2010 as the instruments room at GL was being restored in preparation for the arrival of new equipment funded by the European Regional Development Fund (ERDF 078 - Upgrading of Giordan Lighthouse GAW Research Station). When compared to the other variables, the AP records are available only for the span between 2002 and 2008 when the 21X logger was operating. Following the installation of the new equipment, the AP data was no longer recorded due to the limited analogue channel of the logger provided by the supplier. Taking into account measurement errors and system interruptions, each variable contains the following fractions of the 26-year dataset: 72.3% for AT; 70.86% for DT; 71.8% for RH; 18.5% for AP; 67.4% for WS; and 78.3% for WD. Given the small AP dataset, we have not explored the climatology of this parameter within the context of this study, however, analogous plots with this variable can be found together with the scripts (see Supplementary Images and Software availability).

## Methods

The climate assessment of this meteorological dataset involves a comprehensive analysis using various tools and indices to capture the characteristics of the recorded variables. This section details the methodologies and indices employed to evaluate the climate conditions over the 26-year period.

To thoroughly analyse the dataset, a range of analytical tools was applied. Probability Distribution Functions (PDFs) were constructed from the hourly data to understand the distribution and variability of each meteorological variable. The means, standard deviation, maxima and minima for each of the daily 24-hours were used to construct diurnal cycles. The same statistics were obtained for every day of the year to create annual cycles, illustrating the seasonal tendencies and long-term variations throughout the year. These cycles are essential for identifying recurring patterns and potential anomalies in the data.

Seasonal Wind Roses were created to visualise the predominant wind patterns and variations across different seasons, aiding in understanding the local wind climatology. Finally, time series heat maps were prepared to represent the monthly means of each variable across the entire dataset. These visualisations facilitate the identification of tendencies and anomalies in the data over extended periods, making it easier to spot changes in climate conditions.

To further describe the climatic conditions, we employed a few climate indices ( Dunn *et al.*, 2020; Yu & Zhong, 2019) that provide a deeper understanding of specific weather patterns:

- Summer Days (SU), which accounts for the number of days in a month when the daily maximum temperature (TX) exceeds 25°C;
- Tropical Nights (TR), which measures the number of days in a month when the daily minimum temperature (TN) exceeds 20°C;
- Days exceeding 35°C (TX35), which quantifies the number of days in a month when TX exceeds 35°C;
- Monthly maximum for TX (TXX);
- Monthly minimum for TN (TNN);
- Windy Days (WI), which is a count of the number of days in a month when the mean WS is equal to or greater than Beaufort scale 6 (10.8 m/s or 22 knots).

The SU and TR are indicative of warm weather periods and are useful for assessing the frequency and distribution of hot days and nights over the years, which can have important implications for energy consumption, human comfort, and local ecosystems. TX35 complements these indices by quantifying the occurrence of extremely hot days within each month. Additionally, TXX and TNN provide insight into temperature extremes that stress the limits of the temperature at the station. Finally, the frequency of WI is crucial for understanding wind patterns and their potential impacts on local weather, infrastructure, and activities. The Supplementary Material also includes assessments for the following additional temperature indices: monthly mean for TX (TXm), monthly mean for TN (TNm), Diurnal Temperature Range (DTR; monthly average difference between TX and TN), and Extreme Temperature Range (ETR; difference between TXX and TNN).

To construct the annual cycles for these indices, the original monthly definition of each index was retained as “number of days per month”, but a 30-day running mean (maximum and minimum for TXX and TNN respectively) was applied across the daily time axis. This corresponds to 336 overlapping 30-day windows per year, rather than 12 non-overlapping calendar months, and provides a smoother and more continuous representation of the annual cycle. This approach preserves the index definition and presents a result that is comparable to the other annual cycles presented, while offering enhanced interpretability and robustness.

To investigate long-term trends, we also constructed time series of annual means. However, due to the uneven data coverage across the full record (as shown in Figure 2), certain years may be misrepresented, particularly where seasonal gaps are present. To address this, we applied two filtering thresholds to the annual time series: one including only years with at least 60% data availability, and another using a stricter 80% threshold. The resulting trends proved highly sensitive to the chosen filter. For example, the heat maps reveal that missing summer data in 2014, 2017, and 2022 substantially affects indices such as SU and TR, which rely heavily on warm-season observations. Notably, the trend in TR appears negative when using the 60% filter, but becomes positive when applying the 80% filter. The latter outcome is consistent with broader European and Mediterranean trends in tropical nights ( Correa *et al*., 2024; Klok *et al*., 2023; Yavaşlı & Erlat, 2024). Given these uncertainties, we have chosen not to present trend values in the main text, however, these annual time series plots are included in the Supplementary Material, and relevant observations are mentioned in the main discussion where appropriate. These tools and indices provide a robust framework for assessing the climate conditions represented in the dataset. The subsequent sections will present detailed analyses based on these methodologies, offering insights into the temporal and spatial variations in the recorded meteorological variables.

## Climate assessment

In this section, we present a detailed analysis of the climate conditions captured in our meteorological dataset. The assessment focuses primarily on temperature, humidity, and winds. By examining these key variables separately, we aim to provide a comprehensive understanding of the climate described by the recorded data.

Similar plots and detailed analyses for all other variables, including AP, are available in the data repository alongside the dataset. These additional resources provide a more extensive view of the climate conditions at the station and support the findings discussed in this section.

To better understand the drivers of inter-annual variability influencing the GL station, we investigated correlations between monthly averages of the observed variables and a set of well-established large-scale climate indices. These included the El Niño–Southern Oscillation (ENSO; Kousky & Higgins, 2007; Rasmusson & Carpenter, 1982), the North Atlantic Oscillation (NAO; Barnston & Livezey, 1987; Chen & van den Dool, 2003; van den Dool *et al*., 2000), and the Mediterranean Oscillation Index (MOI; Conte *et al*., 1989; Palutikof, 2003; Palutikof *et al*., 1996). These indices were obtained from the National Oceanic and Atmospheric Administration (NOAA) National Centers for Environmental Information (NCEI), the NOAA Climate Prediction Center (CPC), and the University of East Anglia (UEA) Climatic Research Unity (CRU) respectively.

The correlations, summarized in Table 2, reveal a clear link between the NAO and several climate parameters, most notably AT and WS, consistent with known effects of this teleconnection. The MOI also shows moderate correlations with WD, likely reflecting the influence of regional circulation patterns in the Mediterranean Basin. ENSO-related indices are generally weakly correlated with the local variables, although WS exhibits some sensitivity.

**Table 2.** Pearson correlation between GL variables and three teleconnection indices. ENSO is represented by NINO3.4, and MOI by MOI1. Bold values indicate correlations significant at the 95% confidence level. Correlations for air pressure (AP) are presented for completeness but should be interpreted cautiously due to limited data availability (see Figure 2).

	ENSO	NAO	MOI
**WD**	0.032121	-0.064030	**0.364481**
**WS**	**-0.223180**	**0.361187**	**-0.490180**
**RH**	-0.068790	0.056916	-0.063890
**AT**	0.003897	**-0.384720**	**0.455612**
**DT**	-0.027480	**-0.353610**	**0.423142**
**AP**	-0.018550	**0.243380**	0.038302

Overall, this analysis supports previous findings in the literature (e.g., Brönnimann, 2007; Ciarlo` & Aquilina, 2015; Wallace & Gutzler, 1981) linking Central Mediterranean climate variability to large-scale atmospheric teleconnections. A more comprehensive treatment of this topic, including lagged effects and sub-seasonal dynamics, could be explored in future work. Full correlation matrices and variations of these indices are available in the Supplementary Material.

### Temperature

**Figure 3.**
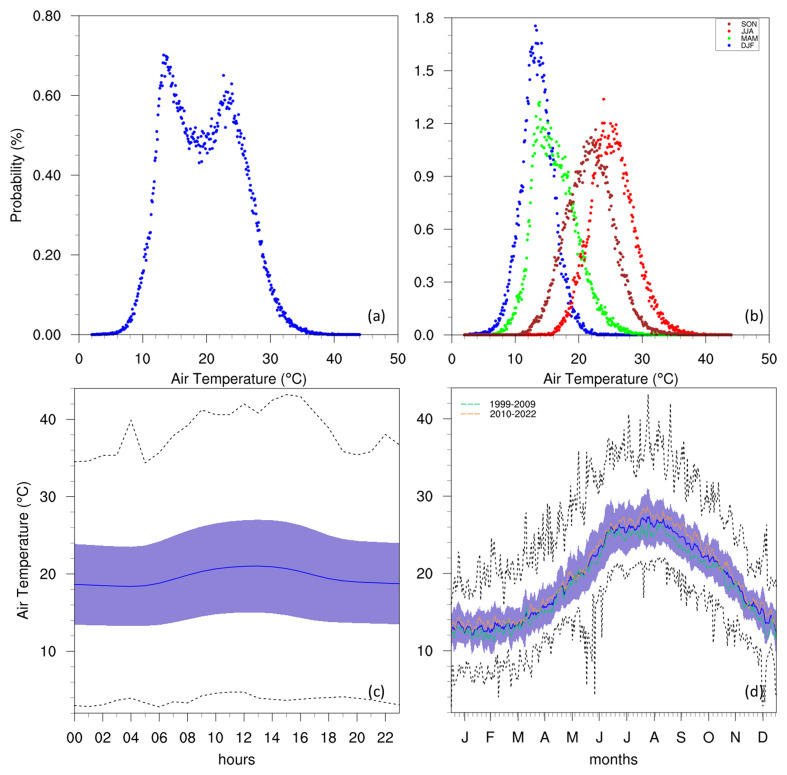
( **a**/ **b**) Annual/seasonal PDFs, ( **c**) diurnal and ( **d**) annual cycles with data distribution for AT. For the seasonal PDFs ( **b**), blue, green, red, brown-red dots represent DJF, MAM, JJA, SON respectively. For the diurnal ( **c**) and annual cycles ( **d**), the solid blue line represents the mean, the shaded area describes one standard deviation above and below the mean (2 sigma), and the dotted black line shows the upper and lower limits. The dashed lines in green and orange ( **d**) represent the means for 1999–2009 and 2010–2022 respectively.

Temperature is a fundamental climate variable, influencing many aspects of the environment and human activities. Figure 3 illustrates this variable at the Giordan Lighthouse in Gozo through a PDF, diurnal cycle, and annual cycle. The PDF ( Figure 3a) shows a clear bimodal distribution for temperature, peaking at around 10 °C and 25 °C, which suggests that the climate in the region fluctuates predominantly between two states: a cool period and a warmer period. This can be seen in more detail when assessing the PDFs of each season separately ( Figure 3b), where DJF and MAM contribute to the low-temperature peak, while JJA and SON contribute to the warmer peak. This does not suggest that the local climate is made up of only two-seasons, as precipitation plays an important role in each season, and the GL data does not include this variable. The annual cycle ( Figure 3d) also describes a typical Mediterranean seasonal cycle, with hot summers peaking in July and August, and cool winters around January and February. Furthermore, both the annual and diurnal cycles ( Figure 3c) show that the minimum temperatures can come close to 0 °C but never reach it, while the most extreme warm temperatures can approach 40 °C in summer and on rare occasions exceed it.

**Figure 4.**
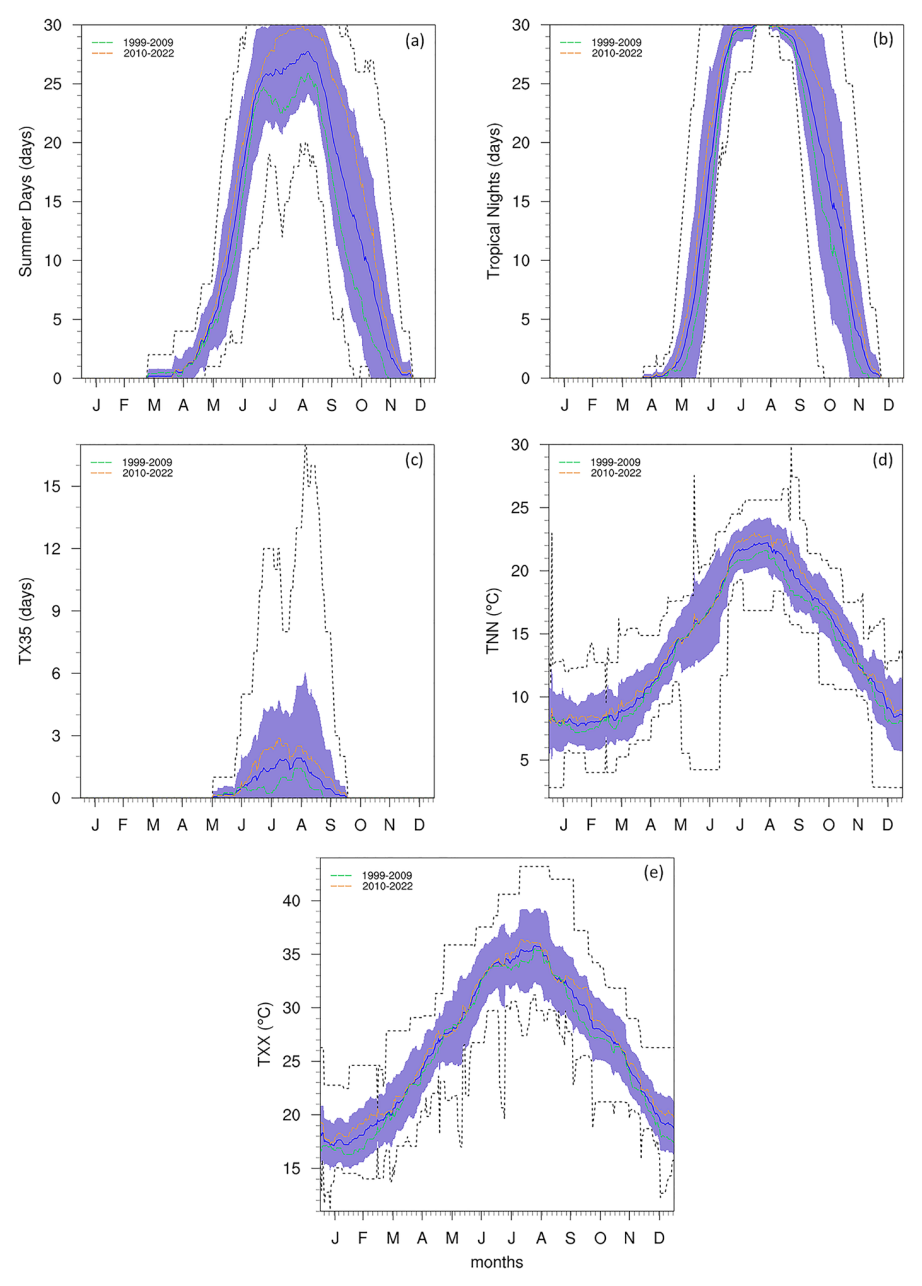
Annual cycle for ( **a**) SU, ( **b**) TR, ( **c**) TX35, ( **d**) TNN, and ( **e**) TXX, TR at the Giordan Lighthouse in Gozo. The solid blue line represents the mean, the shaded area describes one standard deviation above and below the mean (2 sigma), and the dotted black line shows the upper and lower limits. The dashed lines in green and orange ( **d**) represent the means for 1999–2009 and 2010–2022 respectively.

The frequency of warmer and hotter events during the summer period is shown to be very high when looking at the annual cycle of extreme indices ( Figure 4a–b). As the images shown are making use of a 30-day running window for total events, the maximum possible value for SU and TR is 30 days. This maximal value is found within 1 standard deviation of the mean between July and August for SU, and between June and September for TR. This broader peak for TR could explain the rapid rise in temperature prior to the summer months and the slower rate of cooling after. Furthermore, between the end of July and beginning of August, TR is always maximal, indicating this as the most intense warming period of the year.

**Figure 5.**
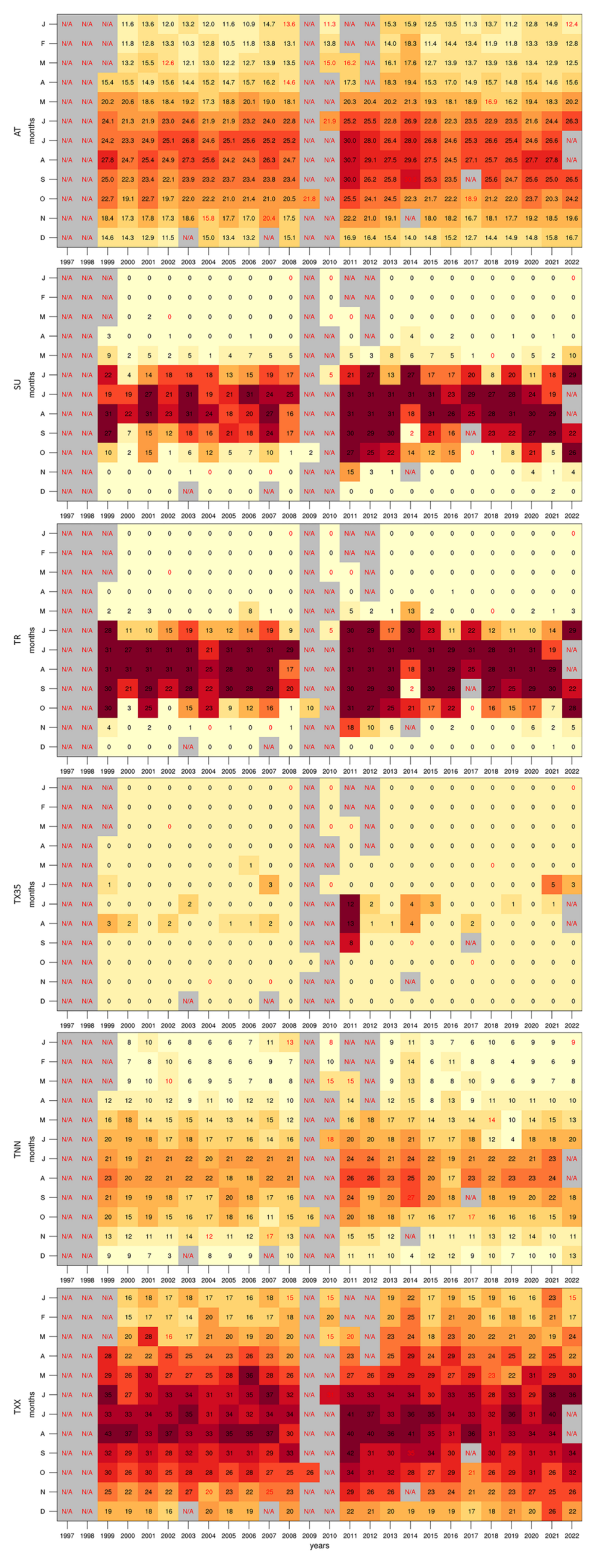
Monthly mean time series heat maps for AT, SU, TR, TX35, TNN, and TXX. The red text describes months with under 40% valid values.

The TX35 ( Figure 4c) displays a distinct seasonal pattern, with frequency peaking sharply in July and August, consistent with the climatological warmest period, and no occurrences prior to May or after September. Notably, TX35 indicates a recurring intensification of extreme heat events in September, suggesting a seasonal extension of summer-like conditions into early autumn. Furthermore, the upper bound of the distribution shows evidence of an anomalous event in which monthly counts exceeded 10 days (this is discussed in more detail in the section describing Figure 5). Complementing this, TXX ( Figure 4e) and TNN ( Figure 4d) highlight the seasonal variations recorded at the station. As expected, the highest TXX values occur in July and August, while the lowest TNN values are recorded between December and January.

Considering the gaps in the dataset, plotting annual means to follow the rate of warming detected by the station could be misleading. However, this can be circumvented with the use of time series heat maps describing monthly means ( Figure 5). This reveals a clear difference between the two decades portrayed, as temperatures (especially summer) in the 2010s become visibly warmer than those in the 2000s. This can also be observed in the annual cycles ( Figure 3 and Figure 4) which show the means for these two time periods. A similar rise in temperature can be seen in the time series of the data measured at the Malta International Airport Meteorological Office ( Galdies, 2022), which shows a gradual rise in the 2000s, with more consistently high annual means after 2010.

### Humidity

**Figure 6.**
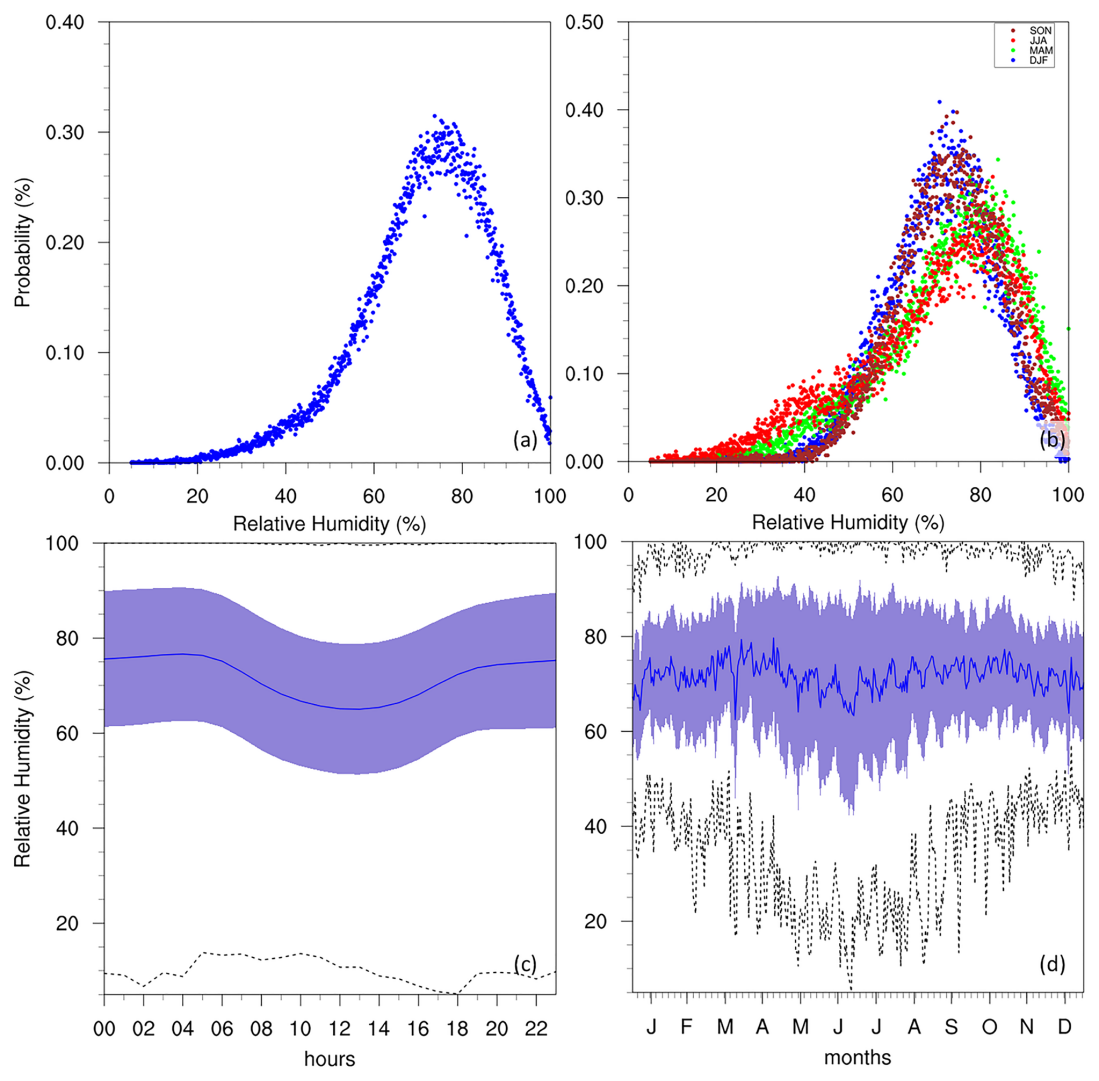
( **a**/ **b**) Annual/seasonal PDFs, ( **c**) diurnal and ( **d**) annual cycles for RH at GL. For the seasonal PDFs ( **b**), blue, green, red, brown-red dots represent DJF, MAM, JJA, SON respectively. For the diurnal ( **c**) and annual cycles ( **d**), the solid blue line represents the mean, the shaded area describes one standard deviation above and below the mean (2 sigma), and the dotted black line shows the upper and lower limits.

According to past studies ( Galdies, 2011; Galdies, 2022), the RH of the Maltese islands is typically between 70 and 80%. The data from the Giordan Lighthouse ( Figure 6) is in agreement with these past studies, as the PDF of hourly RH peaks just below the 80%, and dips during the warmest times of the day (as described in Galdies, 2011) as the capacity of air parcels to hold water increases. The PDF peaks for JJA and SON are very similar, each occurring above 70%, while those for DJF and MAM peak at a slightly higher RH, around 80%. This seasonal pattern aligns with the temperature variability ( Figure 3b) and may also reflect underlying rainfall seasonality (although precipitation data is not available at the station to confirm this directly). A notable dip in average RH in summer can also be observed, however, the standard deviation and limits of RH during this time also broaden, reaching an absolute minimum close to 10%. This is possibly linked to the reduced precipitation of these months (noted by Galdies, 2011; Galdies, 2022). Although evaporation rates would theoretically increase during these periods, this likely does not compensate for the reduction in ambient moisture due to the lack of rainfall. As noted by Galdies (2022), the annual mean time series also show a gradual decrease in RH over time (refer to Supplementary Material, although the magnitude of this trend depends on the data completeness filter applied), a result consistent with expectations under increased warming.

### Winds

**Figure 7.**
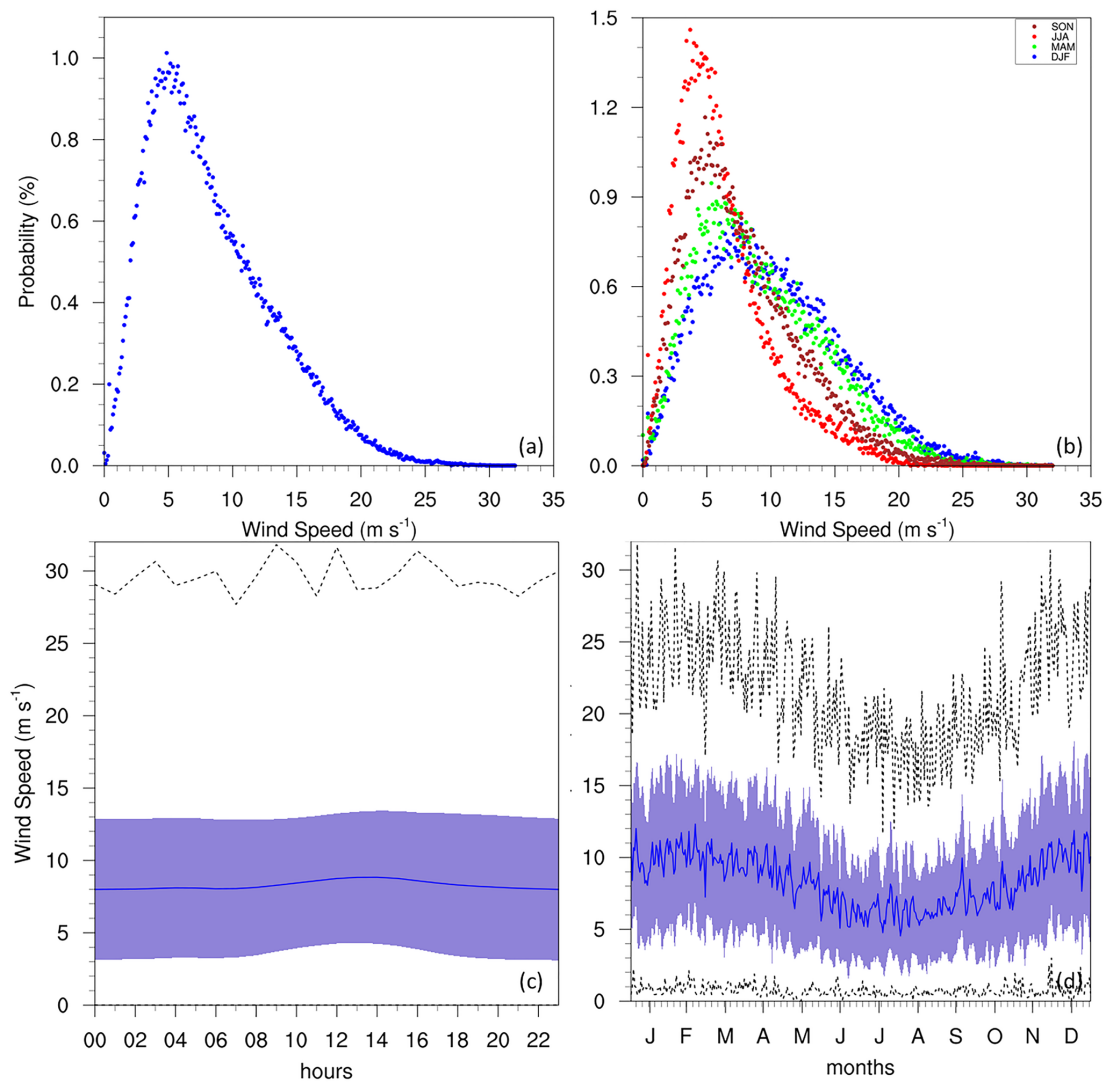
( **a**/ **b**) Annual/seasonal PDFs, ( **c**) diurnal and ( **d**) annual cycles for WS at GL. For the seasonal PDFs ( **b**), blue, green, red, brown-red dots represent DJF, MAM, JJA, SON respectively. For the diurnal ( **c**) and annual cycles ( **d**), the solid blue line represents the mean, the shaded area describes one standard deviation above and below the mean (2 sigma), and the dotted black line shows the upper and lower limits.

**Figure 8.**
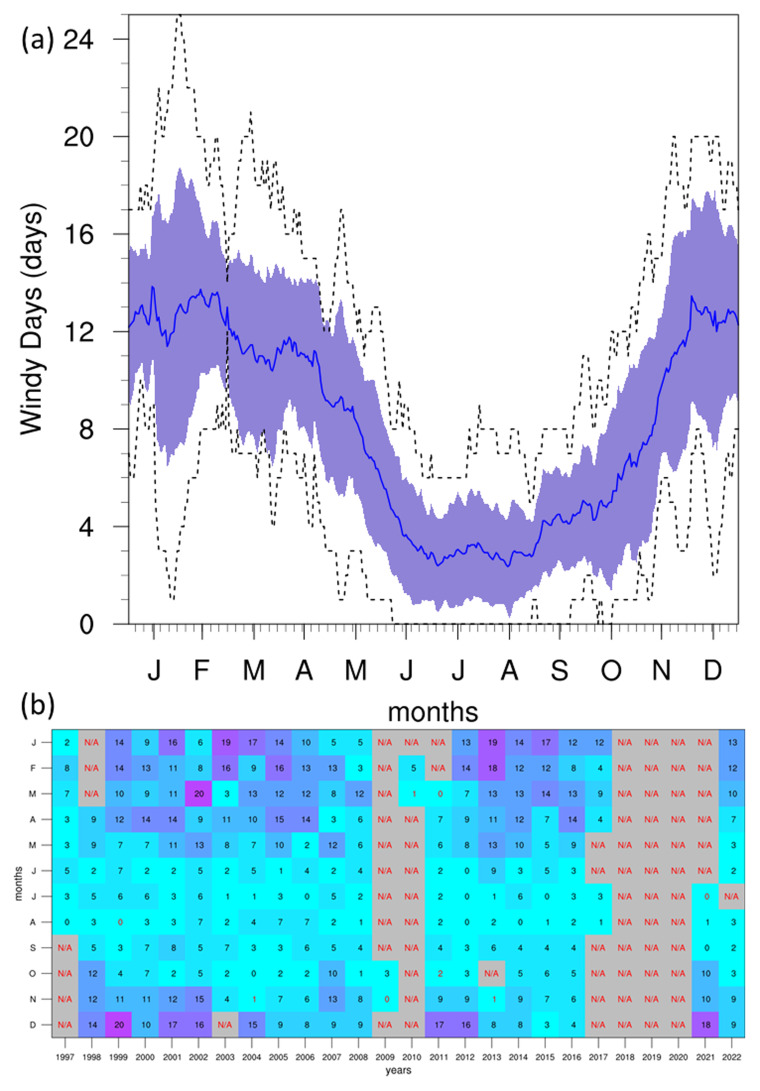
( **a**) Annual cycle and ( **b**) monthly time series heat map for WI at GL. For the annual cycle ( **a**), the solid blue line represents the mean, the shaded area describes one standard deviation above and below the mean (2 sigma), and the dotted black line shows the upper and lower limits. The red text in the time series heat map ( **b**) describes months with under 40% valid values.

**Figure 9.**
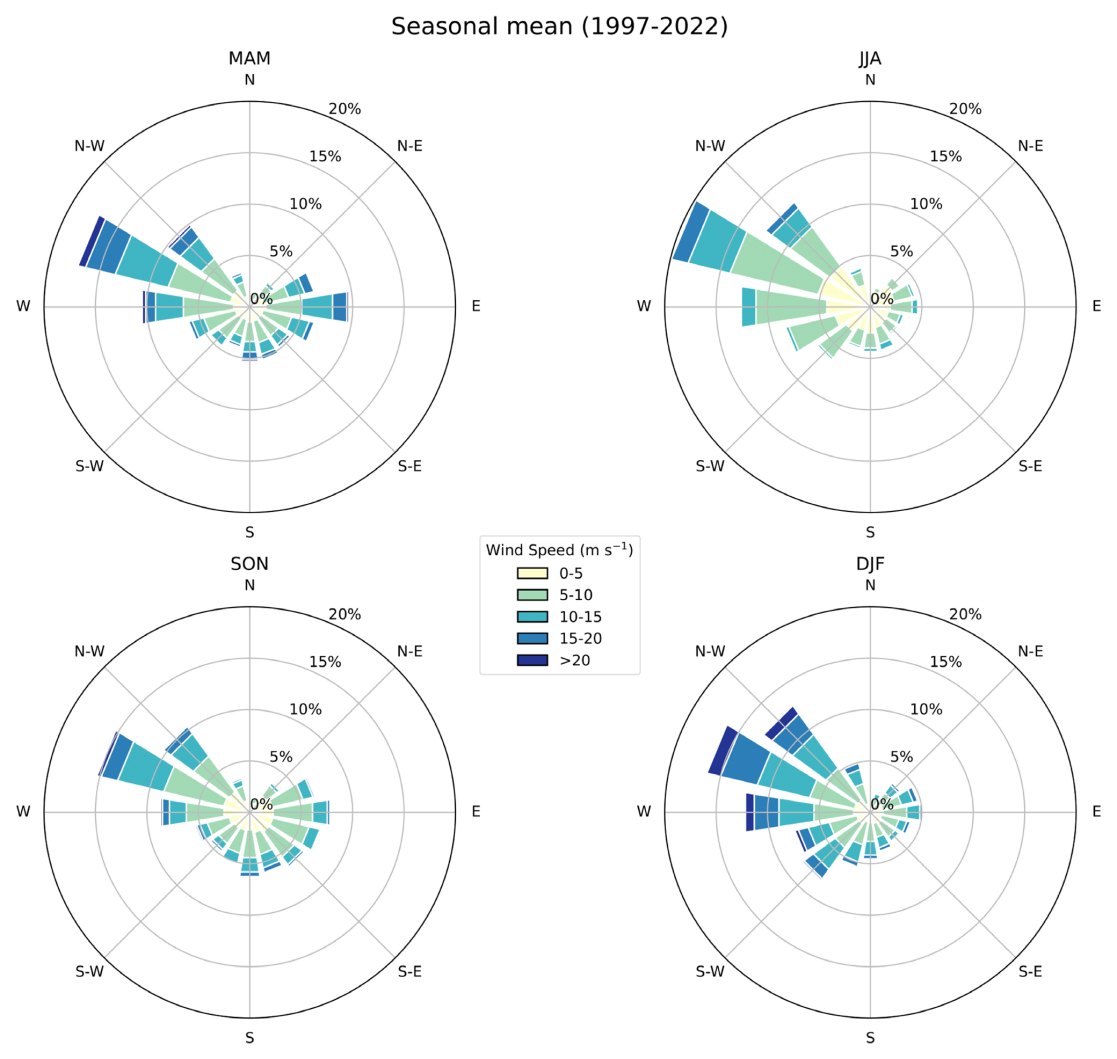
Seasonal wind rose showing WS distribution (m s-1) and frequency (%) at GL. The radial spokes indicate direction, while the length of each spoke corresponds to the frequency of winds coming from that direction. Different colours along the spokes represent varying WS ranges. Labels indicate the seasons: MAM, JJA, SON, DJF.

The WS of the Maltese islands is documented ( Galdies, 2011; Galdies, 2022) at an average of 5.14 m/s (10 knots), corresponding to the most frequent wind conditions at 5 m/s ( Figure 7). The average however, noted more prominently in the diurnal and annual cycles, is shown to be closer to 7 m/s, even reaching 10 m/s during the winter seasons. This could be attributed to the position of the GL at the NW edge of the Maltese islands, combined with the direction of the prevailing winds (discussed below), sea or land breezes, and possibly thermal upslope winds. Another explanation could be related to the site’s exposure to more frequent storms in the Sicily Channel ( Alhammoud *et al.*, 2014; Nissen *et al.*, 2010) that tend to impact the GL more frequently, while the Luqa station would not be impacted by these events as often. The seasonal PDFs also show the probability of more intense wind activity increasing notably during MAM and DJF. However, attributing this feature to specific physical processes requires more targeted investigation beyond the scope of this study. The annual cycle reveals the lowest WS during summer months, with the highest values in winter. This corresponds to the peak of WI ( Figure 8) at approximately 13 days/month in winter, which shows no notable changes in tendency throughout the time-period of the dataset. This high intensity of winds is also notable in the seasonal wind roses ( Figure 9), where the strongest winds occur in the winter months, and the prevailing winds come from the W-NW direction. The frequency of WI may also be declining (see Supplementary Material), suggesting that terrestrial stilling persists on the Maltese Islands ( Galdies, 2022; Wadsworth, 1928), but may also be linked to a decrease in the frequency of cyclonic storms in the region ( Flaounas *et al.*, 2022).

## Summary

This study provides a detailed analysis of the meteorological data collected from the GL in Gozo over a 26-year period from 1997 to 2022. The data includes six key variables: AT, DT, RH, AP, WS, and WD, all recorded with hourly resolution. The analysis aimed to assess the climate variability of the Maltese Islands, emphasising the completeness of the dataset, the characteristics of each variable, and the implications of observed tendencies.

Initially, the dataset's completeness was evaluated, revealing gaps and outliers primarily due to technical issues with the instruments. Notably, AP and WS data had subtantial gaps, and some anomalies were detected which were subsequently excluded from the analysis.

The analysis of temperature data indicated a clear bimodal distribution with peaks corresponding to winter and summer seasons. The annual cycle displayed a typical Mediterranean pattern with hot summers and mild winters. The frequency of warm events, as indicated by the SU and TR indices, showed a high occurrence during summer months, especially in the 2010s, suggesting a warming tendency over the study period.

RH data aligned with previous studies, showing typical values between 70% and 80%, with notable dips during summer months. The variability in humidity was attributed to seasonal changes in temperature and precipitation.

WS analysis revealed that the most frequent wind conditions were around 5 m/s, with a slight peak during midday possibly due to local topographical influences. The annual cycle showed higher WS during winter, consistent with the peak in the WI index. Seasonal wind roses highlighted the predominant wind direction from the W-NW and stronger winds in winter.

This comprehensive assessment of the GL meteorological dataset underscores the importance of long-term monitoring for understanding climate variability. The findings indicate the presence of seasonal patterns and tendencies in temperature, humidity, and wind characteristics over the 26-year period. Notably, the warming tendency observed in the frequency of hot days and nights during the summer months aligns with broader global climate change patterns.

This study highlights the need for continuous and accurate meteorological data collection to monitor and predict climatic changes. The insights gained from the GL dataset contribute to the broader understanding of the Maltese Islands' climate and underscore the value of background monitoring stations in climate research.

Future work should focus on improving instrument reliability (and hence reduce the occurrence of new data gaps), and expanding the analysis to compare to other datasets within the Maltese islands. This will enhance the robustness of climate assessments and support more effective climate adaptation and mitigation strategies for the region.

## Ethics and consent

Ethical approval and consent were not required.

## Data Availability

Zenodo: Giordan Climate Assessment Data & SI, 10.5281/zenodo.14415659 ( Ciarlo` *et al.*, 2024). This study contains the following underlying data: Giordan_data_hr.csv The data is available under Creative Commons Zero v1.0 Universal. Zenodo: Giordan Climate Assessment Data & SI, 10.5281/zenodo.14415659 ( Ciarlo` *et al.*, 2024). This study contains the following underlying data: Figure Legends - *For diurnal and annual cycles the solid blue line represents the mean, the shaded area describes one standard deviation above and below the mean (2 sigma), and the dotted black line shows the upper and lower limits. Dashed lines in green and orange represent the means for 1999–2009 and 2010–2022 respectively.* *For seasonal PDFs, blue, green, red, brown-red dots represent DJF, MAM, JJA, SON respectively.* *Monthly mean time series heat maps use red text for months with under 40% valid values.* *For wind rose, radial spokes indicate direction, while the length of each spoke corresponds to the frequency of winds coming from that direction. Different colours along the spokes represent varying WS ranges. Labels indicate the seasons: MAM, JJA, SON, DJF.* The data is available under Creative Commons Zero v1.0 Universal.

## Software availability statement

- Source code available from: https://github.com/ciarloj/giordan-lighthouse-climate-assessment
  License: CC0 - 1.0
- Archived software available from: https://doi.org/10.5281/zenodo.14415659 ( Ciarlo` *et al.*, 2024)
  License: Creative Commons Zero v1.0 Universal
