# Supplementary material for: Insights into climate variability of the meteorological records from a background monitoring station: the Giordan lighthouse, Gozo
Source: Open Res Eur. 2025 Jul 15;5:37. Originally published 2025 Feb 3. [Version 2] doi: 10.12688/openreseurope.19099.2 (PMC12361886; doi:10.12688/openreseurope.19099.2)
Supplement: Peer review history

# Reviewer report: Reviewer response for version 2

**DOI:** 10.21956/openreseurope.22677.r56746

**Published:** 2025-08-22

**Authors:** Pavlos Kalabokas

**Affiliations:** 1 Research Center for Atmospheric Physics and Climatology, Academy of Athens, Athens, Greece

I would like to inform you that the authors have addressed properly our remarks and introduced the requested changes, so we find that the manuscript is now ready for indexing.

Is the study design appropriate and does the work have academic merit?

Yes

Is the work clearly and accurately presented and does it cite the current literature?

Yes

If applicable, is the statistical analysis and its interpretation appropriate?

Partly

Are all the source data underlying the results available to ensure full reproducibility?

Yes

Are the conclusions drawn adequately supported by the results?

Yes

Are sufficient details of methods and analysis provided to allow replication by others?

Yes

Reviewer Expertise:

NA

I confirm that I have read this submission and believe that I have an appropriate level of expertise to confirm that it is of an acceptable scientific standard.

# Reviewer report: Reviewer response for version 2

**DOI:** 10.21956/openreseurope.22677.r56745

**Published:** 2025-08-18

**Authors:** Paolo Cristofanelli

**Affiliations:** 1 Institute of Atmospheric Sciences and Climate (ISAC), Bologna, Italy

The authors addressed all my comments and I would recommend the indexing of the manuscript.

Is the study design appropriate and does the work have academic merit?

Yes

Is the work clearly and accurately presented and does it cite the current literature?

Yes

If applicable, is the statistical analysis and its interpretation appropriate?

Partly

Are all the source data underlying the results available to ensure full reproducibility?

Yes

Are the conclusions drawn adequately supported by the results?

Partly

Are sufficient details of methods and analysis provided to allow replication by others?

Yes

Reviewer Expertise:

Atmospheric sciences

I confirm that I have read this submission and believe that I have an appropriate level of expertise to confirm that it is of an acceptable scientific standard.

# Reviewer report: Reviewer response for version 1

**DOI:** 10.21956/openreseurope.20670.r51836

**Published:** 2025-04-14

**Authors:** Giandomenico Pace, Virginia Ciardini

**Affiliations:** 1 Energy and Sustainable Economic Development, ENEA - Italian National Agency for New Technologies, Rome, Italy; 2 Department for Sustainability, ENEA National Agency for New Technologies Energy and Economic Sustainable Development, Rome, Italy

The manuscript of Ciarlò et al. presents and discusses a series of approximately 25 years (1997-2022) of meteorological data collected at the Jordan Lighthouse Background Monitoring Station that has been part of the Global Atmosphere Watch (GAW) since 2005.

 After a brief description of the instrumentation and the data selection methodology, the metrics used to discuss the variability and patterns in the database are introduced. Due to several interruptions, the atmospheric pressure time series is not analysed, while temperature, relative humidity and wind behavior is discussed separately mainly in terms of probability distribution, daily and seasonal variability. The analysis also includes the discussion of three indices such as summer days, tropical nights and windy days. The conclusions, agree with other work on the Maltese Islands, show a bimodal annual distribution of daily temperatures, the increase in the average daily temperature during the 2020s decade compared to that measured in 2010s associated to an increase in the number of summer days and tropical nights. Relative humidity shows limited variability throughout the year and a daily minimum around the hottest hours of the day. The distribution of winds is dominated by a W-NW direction and a greater occurrence of winds around 7 m/s. An increase in the average daily value around the hottest hours is suggested to be due to anabatic winds connected with the location of the Station on the top of a hill.

 The manuscript is clear in both description and purpose and the results are consistent with those of previous studies. The use of precipitation and atmospheric pressure data would certainly have extended the conclusions and expanded the authors' ability to provide a more original discussion.

 Overall, it provides a useful contribution to the climatological characterization of the central Mediterranean, even if the analysis would have been more complete, for example by trying to investigate the annual variability as a function of indices such as North Atlantic Oscillation, Mediterranean Oscillation or El Niño/Southern Oscillation.

 In our opinion, the work would be more complete by clarifying and/or adding some information on the following points:

 - In the 25 years of observations, the replacement of the anemometer and the use of the same humidity and temperature sensor is reported (p. 4). What is missing, however, is any information on the maintenance and recalibration of the instrumentation used during the period as well as more details in the description of the data quality control procedure. The authors stated that they removed data points due to problems identified with the instruments; we suggest to add information about the whole data quality control procedure (e.g. control algorithm, manual check, etc.) and briefly reporting on maintenance and calibration checks.

 - In the text it is clear that data are sampled every hour (first paragraph p. 5), but it is not specified whether the hourly data corresponds to instantaneous values or average values.

 -  p. 5 “To construct the  annual  cycles  for  these  indices,  a  30-day   running   average was used to obtain a “daily” frequency comparable to other annual cycles.” Is it correct that this sentence only refers to the averages shown in Figures 4(a)-(b) and 8(a)? The other average behaviors (Figures 3 (c)-(d), 6 (b)-(c), 7(c)) are calculated by averaging the average values of each individual days of the year; is this correct?

 - p.5 e 6.: On pp. 5 and 6, the authors report specific instances of outliers in the text; we suggest to collect them in a table providing a more schematic and readable information to the readers.

 - p.8: Comment to figure 3 c, d : “Furthermore, both the annual and diurnal cycles ( Figure 3c) show that the minimum temperatures often come close to 0 °C but never reach it, while the most extreme warm temperatures can approach 40 °C in summer and on rare occasions exceed it.” Please replace “often” with “can”

 - For the reader, it would be clearer the meaning of Figures 2, 5, 8 (b) if color scales linked to the occurrence of the event were shown.

 - For greater uniformity of the results shown, similarly to figure 3 (temperature behavior), I would also show both the seasonal and annual distribution in figures 6 and 7, showing the seasonal distribution of RH and wind intensity respectively.

 - Looking at figure (8)b one gets the impression that the number of windy days has slightly decreased over the years. Averaging over the number of windy days yearly, from June to June, is there any decrease? Although the wind measurement was extensively discontinued from 2017 to 2021, it would be interesting if there was a trace of this behaviour.

 - Page 15 ‘The findings indicate significant seasonal patterns and trends in temperature, humidity, and wind characteristics over the 26-year period.‘ I would reformulate the sentence by removing the word trend since only temperature shows a clear increase in the database and no value was derived that could be associated with an annual trend (e.g. annual increase of the selected variable).

 - Reference list: please check links to “Reference Source” ( www.um.edu.mt) that do not seem to work.

Is the study design appropriate and does the work have academic merit?

Yes

Is the work clearly and accurately presented and does it cite the current literature?

Yes

If applicable, is the statistical analysis and its interpretation appropriate?

Yes

Are all the source data underlying the results available to ensure full reproducibility?

Yes

Are the conclusions drawn adequately supported by the results?

Yes

Are sufficient details of methods and analysis provided to allow replication by others?

Partly

Reviewer Expertise:

data analysis, interaction between meteorological parameters, aerosols, clouds and short- and long-wave radiation

We confirm that we have read this submission and believe that we have an appropriate level of expertise to confirm that it is of an acceptable scientific standard, however we have significant reservations, as outlined above.

# Response

**Published:** 2025-07-11

**Authors:** James Ciarlo

**Affiliations:** University of Malta, Msida, Malta

We are sincerely grateful to the team of reviewers for taking the time to carefully evaluate our manuscript and provide thoughtful, constructive feedback. We are confident that their input has significantly improved the quality and clarity of the paper. Please find our detailed responses to each of the comments below.  

*In response to the comment, "The use of precipitation and atmospheric pressure data would certainly have extended the conclusions and expanded the authors' ability to provide a more original discussion.":*

 We agree that including additional variables would have enhanced the analysis. However, we note that the Giordan station does not collect precipitation data. Moreover, due to the limited temporal coverage of the atmospheric pressure dataset, analysis with this data would be less robust than the other variables analysed in the main text. Nevertheless, for completeness and transparency, we have included the corresponding atmospheric pressure plots in the Supplementary Material.

*In response to the comment, "Overall, it provides a useful contribution to the climatological characterization of the central Mediterranean, even if the analysis would have been more complete, for example by trying to investigate the annual variability as a function of indices such as North Atlantic Oscillation, Mediterranean Oscillation or El Niño/Southern Oscillation.":*

 We appreciate the reviewer’s suggestion to further explore the role of large-scale climate modes. In response, we have added a new subsection to the beginning of the "Climate Assessment" section that evaluates the correlation between observed climate variables and teleconnection indices. We agree that further exploration of lagged and nonlinear responses would be valuable and hope to address this in future work. The remaining points presented below are addressed in the same order in which they were raised by the reviewer, to facilitate clarity and ease of cross-referencing.

1. We have added information to the manuscript text describing the maintenance procedures and calibration practices applied throughout the observation period. In addition, Table 1 now includes updated information on the instruments used.
2. The data is sampled every minute and averaged to produce hourly values. This has been clarified in the manuscript.
3. Yes, this is the correct interpretation. In the new manuscript, we have revised and expanded this sentence to improve clarity.
4. These anomalous readings were collected in separate csv files and included with the supplementary material.
5. Completed.
6. We appreciate the reviewer’s suggestion. However, we would like to clarify that Figures 2, 5, and 8(b) are intended as time series heat maps using a linear colour scale applied independently to each panel. The colours are not linked to the occurrence of a specific event but instead serve to highlight the distribution of values. Since the actual values are printed directly on the plots, we opted not to include colour bars to avoid redundancy and to maximise the size and readability of the figures. To improve clarity for the reader, we now refer to these figures explicitly as time series heat maps in the manuscript and have added a clarifying note in the figure captions.
7. We have revised Figures 6 and 7 to include both seasonal and annual distributions of relative humidity and wind intensity, in line with the structure used in Figure 3. Corresponding adjustments have also been made in the text to reflect these changes.
8. Thank you for this observation. The annual mean time series for windy days does indicate a decreasing tendency. However, due to the data discontinuities and the sensitivity of the trend to data availability thresholds, we have not included a quantified trend in the main text. That said, this pattern is now discussed briefly in the manuscript, and the filtered time series plots are available in the supplementary material.
9. Given the issue with quantifying trends, we have changed the terms “trend” throughout the document.
10. All links to the “Reference Source” (www.um.edu.mt) were double-checked and confirmed to be working at the time of submission of the updated manuscript.

# Reviewer report: Reviewer response for version 1

**DOI:** 10.21956/openreseurope.20670.r51837

**Published:** 2025-04-09

**Authors:** Pavlos Kalabokas, John Kapsomenakis

**Affiliations:** 1 Research Center for Atmospheric Physics and Climatology, Academy of Athens, Athens, Greece

The study “Insights into climate variability of the meteorological records from a background monitoring station: the Giordan lighthouse, Gozo,” by Ciarlo et al. provides a valuable analysis of climatological conditions and trends based on data from WMO/GAW regional station at El Gozo (Malta). The manuscript is well-written and clear, though some minor revisions are needed before final acceptance.

- Page 5 (Figure 1). Please add in the map the orography
- The analysis for extreme heat is based on the number of SU (summer days) and TR (tropical nights). I would suggest expanding the analysis including indices for more extreme conditions such as Tx35 (number of days with Tmax exceeding 35 oC).
- In addition, it would suggest investigating if there is a seasonality in the differences between the 2 periods particularly for extreme heat indices. For instance, it seems from figure 4 (but is not so clear) that is a significant increase of TR during transitional seasons indicating an expansion of summer conditions during Spring and Autumn.
- I think that it would be useful to add a paragraph where the climate trends observed in Gozo will be compared with those reported in other regions of the Mediterranean.

Is the study design appropriate and does the work have academic merit?

Yes

Is the work clearly and accurately presented and does it cite the current literature?

Yes

If applicable, is the statistical analysis and its interpretation appropriate?

Partly

Are all the source data underlying the results available to ensure full reproducibility?

Yes

Are the conclusions drawn adequately supported by the results?

Yes

Are sufficient details of methods and analysis provided to allow replication by others?

Yes

Reviewer Expertise:

NA

We confirm that we have read this submission and believe that we have an appropriate level of expertise to confirm that it is of an acceptable scientific standard, however we have significant reservations, as outlined above.

# Response

**Published:** 2025-07-11

**Authors:** James Ciarlo

**Affiliations:** University of Malta, Msida, Malta

We are sincerely grateful to the team of reviewers for taking the time to carefully evaluate our manuscript and provide thoughtful, constructive feedback. We are confident that their input has significantly improved the quality and clarity of the paper. Please find our detailed responses to each of the comments below.   All points below are addressed in the same order in which they were raised by the reviewer, to facilitate clarity and ease of cross-referencing. 

1. *Response to Page 5 (Figure 1):* Figure 1 and the associated caption were updated to include an elevation map. We have modified the image further by including a photo of the station as suggested by another reviewer. We have removed the map of the Mediterranean to allow for a more presentable image, while the location context is still present by showing latitude and longitude. 
2. Thank you for this valuable suggestion. In response, we have expanded the analysis to include TX35, as well as the monthly maximum of daily maximum temperature (TXX) and the monthly minimum of daily minimum temperature (TNN). These additions provide a clearer picture of extreme temperature events and seasonal variability. The associated analysis and figures have been updated accordingly in the main text. Additional indices and detailed results are available in the supplementary material.
3. We have expanded the set of extreme heat indices as suggested, adding TX35, TXX, and TNN. These show a clearer intensification of heat extremes, particularly in September, which may influence patterns during the SON season. Additionally, seasonal PDFs have been added for RH and WI to further support the discussion of seasonal variability. These additions have been implemented and discussed in the revised text.
4. Thank you for the suggestion. While we prepared annual mean time series for key variables, the trends proved highly sensitive to data completeness thresholds and were therefore not included in the main text. Given this uncertainty, we opted not to draw direct comparisons with other Mediterranean regions. However, we have now included this explanation in the manuscript and the filtered plots are available in the supplementary material, and we refer to broader regional patterns where relevant in the discussion.

# Reviewer report: Reviewer response for version 1

**DOI:** 10.21956/openreseurope.20670.r51834

**Published:** 2025-03-26

**Authors:** Paolo Cristofanelli

**Affiliations:** 1 Institute of Atmospheric Sciences and Climate (ISAC), Bologna, Italy

This paper by Ciarlo et al. presents an interesting study on the climate variability of the meteorological records of the WMO/GAW regional station of El Gozo (Malta).

 This work can serve as an updated reference for the characterisation of the meteorological conditions at this important measurement site in the Mediterranean basin, a well-known hotspot for climate change and pollution export from Europe.

 The manuscript is clear and well written, but some minor adjustments should be made before final acceptance.

 1) In the "Methods" section, the authors cite a comparison with results from Malta Airport. However, I could not find a direct comparison of the two datasets in the manuscript. I would suggest to the authors to provide evidence of this comparison or to revise the "Methods" section

 2) page 5 (Figure 1). I would suggest adding an image of the station location

 3) Table 1. It would be useful to add information about the instruments used, the observation periods and the measurement uncertainties

 4) Page 6 (Methods). It's not clear to me why the authors applied a 30-day running mean to the number of SU and TR. According to the reported definitions of SU and TR, these descriptors are "number of days in a month", so how can a 30-day running mean be calculated from a monthly frequency?

 5) Page 9 ("Winds"): To explain the diurnal WS variability, the authors refer to anabatic/katabatic winds. I'm reluctant to believe that the small hill on which the station is located can create the thermal gradient to trigger upslope winds. Besides thermal upslope winds, can sea/land breezes be considered?

 Pag 15 (Summary): As no trend detection or quantification statistical analyses have been carried out, I recommend to avoid mentioning the existence of a "trend", but to talk about "tendencies". Also, when describing long-term trends, avoid terms that imply statistical analysis (i.e. use "apparent" instead of "significant").

Is the study design appropriate and does the work have academic merit?

Yes

Is the work clearly and accurately presented and does it cite the current literature?

Yes

If applicable, is the statistical analysis and its interpretation appropriate?

Partly

Are all the source data underlying the results available to ensure full reproducibility?

Yes

Are the conclusions drawn adequately supported by the results?

Partly

Are sufficient details of methods and analysis provided to allow replication by others?

Yes

Reviewer Expertise:

Atmospheric sciences

I confirm that I have read this submission and believe that I have an appropriate level of expertise to confirm that it is of an acceptable scientific standard, however I have significant reservations, as outlined above.

# Response

**Published:** 2025-07-11

**Authors:** James Ciarlo

**Affiliations:** University of Malta, Msida, Malta

We are sincerely grateful to the team of reviewers for taking the time to carefully evaluate our manuscript and provide thoughtful, constructive feedback. We are confident that their input has significantly improved the quality and clarity of the paper. Please find our detailed responses to each of the comments below.  

1. We are grateful to the reviewer for presenting this point. The “Methods” section in the Abstract was intended to describe a qualitative comparison to other studies (e.g. Galdies, 2022) and not a direct quantitative comparison using the Malta Airport dataset. This sentence was clarified in the manuscript.
2. Figure 1 and the associated caption were updated to include a photo of the station. We have modified the image further by including an elevation map as suggested by another reviewer. We have removed the map of the Mediterranean to allow for a more presentable image, while the location context is still present by showing latitude and longitude. 
3. We have updated Table 1 to include these details.
4. To clarify, we maintained the standard definitions of SU and TR as “number of days per month,” but instead of using 12 discrete monthly values, we calculated a 30-day running mean over the daily time axis, resulting in 336 overlapping windows. This approach does not change the index definitions but provides a smoother depiction of the annual cycle, which is more suitable for comparison with other climatological fields. We have now revised the text in the Methods section to clarify this point.
5. We have revised the manuscript to acknowledge that land/sea breezes could also contribute to the observed diurnal variability in wind speed. These considerations have been added alongside mentions of thermal upslope winds to present a more balanced interpretation.
6. *Response to Summary:* We have changed the terms “trend” and “significant” throughout the document.

# Reviewer report: Reviewer response for version 1

**DOI:** 10.21956/openreseurope.20670.r51383

**Published:** 2025-03-25

**Authors:** Sultan Hameed

**Affiliations:** 1 Stony Brook University, Stony Brook, USA

This paper is potentially a useful contribution by presenting a first analysis of a 26 year long meteorological data set at the Giordan lighthouse in Malta. The results of this analysis can be considered as preliminary because the data have not been subjected to quality testing and adjustment. The manuscript needs some corrections and further analysis, as suggested below, before it can be recommended.

1. The authors removed air pressure readings below 950 hPa without giving a justification for this cut-off. Data at Malta international airport which is only 30 miles away from Gozo can be used as a guide to estimate the lowest air pressure that is likely to be observed at Gozo. Similarly, the lower and upper limits of air temperature can be estimated from the temperature data at the airport.
2. It would be very useful to plot time trends in seasonally averaged temperatures, summer days and tropical nights for 1997-2022.  Such trend figures will quantify the rate of climate warming in Malta.
3. It would also be very useful to calculate correlations between seasonal averages of all the variables in Table 1 with the North Atlantic Oscillation to see how much of the inter-annual variability in these variables can be explained by pressure variations over the North Atlantic.

Is the study design appropriate and does the work have academic merit?

Yes

Is the work clearly and accurately presented and does it cite the current literature?

Yes

If applicable, is the statistical analysis and its interpretation appropriate?

Partly

Are all the source data underlying the results available to ensure full reproducibility?

Yes

Are the conclusions drawn adequately supported by the results?

Partly

Are sufficient details of methods and analysis provided to allow replication by others?

Yes

Reviewer Expertise:

NA

I confirm that I have read this submission and believe that I have an appropriate level of expertise to confirm that it is of an acceptable scientific standard, however I have significant reservations, as outlined above.

# Response

**Published:** 2025-07-11

**Authors:** James Ciarlo

**Affiliations:** University of Malta, Msida, Malta

We are sincerely grateful to the team of reviewers for taking the time to carefully evaluate our manuscript and provide thoughtful, constructive feedback. We are confident that their input has significantly improved the quality and clarity of the paper. Please find our detailed responses to each of the comments below.   We are very grateful for the reviewer’s comment. Following the suggestions of all reviewers, we have applied the recommended changes, and believe this has helped to strengthen the overall quality and reliability of our study.

1. Pressure range from the Malta international airport (MIA) is between 1014.9 to 1018.3 hPa according to Galdies (2010) with no significant changes reported in Galdies (2022). Five instances were identified where the pressure was around 750 hPa, with a change of well over 200 hPa from the previous and following time-steps - the minimum, after excluding these values, was 967 hPa. To remove only the erroneous values, a filter of 950 hPa (an arbitrary value below 967 hPa) was applied. As filter itself is not of import, we have rephrased the explanation to include the specific values removed and why. 
  Similarly, temperature ranges from 1.4 to 43.8 degrees Celsius, according to Galdies (2010), with no precise changes were mentioned in Galdies (2022). A few instances of sudden increases in temperature (a jump of around 20 degrees in the span of an hour) were removed leaving behind a range of 3 to 43 degrees. Once again the filter mentioned was arbitrary to resolve the issue, but does not explain the problem properly. This was corrected in the manuscript. 
2. We agree that long-term trend analysis would provide useful insights. As part of our investigation, we prepared annual mean time series for key variables, however, given the variable data coverage across the observational record, we constructed time series using both 60% and 80% data availability thresholds per year. We found that trend values were highly sensitive to the applied filter. For instance, the trend in TR appears negative under a 60% threshold but turns positive under 80%, in line with broader regional trends. Due to this sensitivity and the potential for misrepresentation, we opted not to include quantified trends and figures in the main text. However, the plots are provided in the supplementary material, and relevant patterns are discussed in the manuscript where appropriate.
3. As recommended, we have carried out correlation analyses between our observed climate variables and the NAO index. Following suggestions from another reviewer, we have also extended the analysis to include other teleconnection indices, including the Mediterranean Oscillation Index and ENSO. A summary of these correlations is now included at the beginning of the "Climate Assessment" section (Table 2). 
